# Supplementary material for: Are carbon emissions trading and green financial instruments synergistic? -Comprehensive quantitative research based on content analysis
Source: PLoS One. 2024 Mar 7;19(3):e0298601. doi: 10.1371/journal.pone.0298601 (PMC10919601; doi:10.1371/journal.pone.0298601)
Supplement: S1 Data — (ZIP) [file pone.0298601.s001.zip › Data/地级市Z_F工作报告.zip!/─┌├╔╣┼/│α╖σ╩╨/└┤╫╘╣1⁄2╓┌║┼í╛┬φ┐╦╩2╛▌═°í┐_2019.docx]

各位代表：

　　现在，我代表市人民政府，向大会报告工作，请予审议，并请市政协委员和其他列席会议的同志提出意见。

　　一、2018年工作回顾

　　过去的一年，市政府坚持以习近平新时代中国特色社会主义思想为指导，深入贯彻落实中央、自治区和市委决策部署，统筹推进“稳增长、促改革、调结构、惠民生、防风险”各项工作，稳中求进、攻坚克难，较好地完成了市七届人大一次会议确定的年度目标任务。预计全市地区生产总值完成1520亿元，增长6%；按照新的统计方式，规模以上固定资产投资增速高于全区平均水平10个百分点；社会消费品零售总额815亿元，增长7.5%；城乡常住居民人均可支配收入分别为31885元和11284元，增长7.5%和9%。一般公共预算收入完成108.2亿元，增长7.5%。主要指标增速均居自治区前列。

　　一年来，我们重点做了以下六个方面工作：

　　（一）着力打好“三大攻坚战”。精准脱贫取得重大突破。牢记总书记“扎实推进脱贫攻坚”的重要指示，聚焦“两不愁三保障”出台政策，加强对基层工作的务实指导，有效压实责任、传导压力。聚焦深度贫困地区强化措施，推出了助力巴林左旗脱贫攻坚的45条意见，同步加大了对全市深度贫困苏木乡镇的扶持力度。聚焦重点难点问题补齐短板，贫困家庭危土房基本清零，健康扶贫兜底保障机制日臻完善，移民搬迁工作进一步规范，资金管理中存在的问题得到及时整改。新一轮京蒙对口帮扶全面加强，实施扶贫协作项目76个，落实资金超过前6年总和。国家安全部、中化集团、国家能源集团、保利集团等中央和国家机关定点扶贫工作成效显著。全市整合投入各类扶贫资金96.8亿元，贫困人口净减少8.14万人，贫困发生率降至1.48%。林西县在全区率先退出国家贫困县序列并荣获全国脱贫攻坚组织创新奖，右旗、喀旗、宁城、松山、克旗5个贫困旗县区接受了自治区脱贫退出第三方考核评估。污染防治成效显著。认真开展中央和自治区环保督察反馈问题整改，集中整治各类问题434个。突出大气、水、土壤污染防治，扎实推进12个专项治理工程，中心城区全年空气质量优良天数达到331天，地表水断面达标率提高至62.5%，土地面源污染得到有效控制。统筹推进生态文明建设，老哈河上游生态保护与环境整治工程完成投资42亿元，新增营造林20.3万亩，退耕还林还湿7万亩，封停中心城区自备水源井126眼，建设污水处理、垃圾治理工程12个。达里诺尔流域生态环境综合治理工程启动实施，年内整合投入资金4700万元。重大风险得到有效防控。认真核实梳理政府债务底数，分类制定政府隐性债务清偿计划，通过政府投资项目“停缓瘦”，压缩一般性支出，扩大政府性基金收入，全年化解政府债务291亿元，超额完成年度任务，“十个全覆盖”十项内拖欠工程款全部化解。积极开展非法集资、农村牧区高利贷、互联网金融风险排查整治，查处案件80起，涉案金额3.5亿元。及时出台了规范房地产市场秩序的24条措施，有效遏制了房价非理性上涨态势，稳定了社会预期。

　　（二）着力推动经济高质量发展。农牧业供给侧结构性改革持续深化。坚持不懈调结构，“增经稳粮、增牛稳羊、增禽稳猪”工程扎实推进，全年压减玉米种植面积55.7万亩，新增设施农业20万亩、优质紫花苜蓿20万亩，肉牛存栏达到161万头，家禽养殖量达到8000万羽。坚持不懈转方式，严控新增水浇地，新建高效节水灌溉40万亩，新增市级以上产业化重点龙头企业24家、农牧业新型经营主体888家。坚持不懈促融合，新建田园综合体21个，宁城县被自治区确定为一二三产业融合发展先导区。坚持不懈创品牌，全市新增“三品一标”认证农畜产品67个。加强动物疫病防控工作，年内没有发生重大动物疫情。工业集中集约集聚发展效果初显。举全市之力打造赤峰东部工业走廊，完成基础设施投资21.9亿元，“七通一平”基本实现，高新区正在成为全市工业经济高质量发展的核心平台，旗县区工业园区整合压缩为13个，优化布局迈出了实质性步伐。云铜40万吨铜冶炼技改升级项目具备试车点火条件，金剑40万吨铜冶炼技改升级项目开工建设，东岳氢氟酸等3个“飞地”项目签约落地。普因医药产业化、泰领海创生物科技园等项目加快建设，富勒烯产业化、拓佳电子产业园等项目加快推进，阿旗凌云海制糖、翁旗凌志薯条加工、林西恒光大中药材深加工等一批项目建成。预计全市规模以上工业增加值增长9%，工业企业利润增长10%。服务业新的增长点正在形成。旅游业突出提高基础保障水平，开工建设旅游公路14条、特色旅游小镇7个、旅游厕所160个，一批重点旅游项目投入运营，克什克腾石阵晋升为国家5A级景区，全市游客人数和旅游收入分别增长13%和16.7%。商贸物流业突出打造新平台新业态，新型基层供销合作体系日益完善，维多利摩尔城、桥北新众联、龙腾国际等项目加快推进，利丰汽车市场完成退城入园，蒙东医药物流园、蒙东峰创、盘古电商园等新型物流平台投入运营，全市物流业实现销售收入550亿元，电商交易额达到28亿元。金融业突出增强服务能力，全市金融机构存贷款余额2310.4亿元和1868.5亿元，增长6.9%和13%，塞北金融小镇投入运营，签约入驻企业64家，华夏银行入驻赤峰，新增资本市场挂牌中小企业53家。

　　（三）着力提高改革开放水平。重点领域改革纵深推进。41项年度改革任务全面完成，35项持续推进的改革任务取得阶段性成果。深入实施生态文明体制改革，市县乡村四级河湖长制全面推行，生态保护红线基本划定。大力推进农村牧区综合改革，国有林场改革全面完成，土地草牧场确权工作基本完成，农村牧区集体产权制度改革、农业水价综合改革试点工作取得积极进展。统筹推进社会事业改革，务实出台医药卫生体制改革23条意见，组建医联体33个，家庭医生签约服务、分级诊疗制度初步建立；4项教育改革创新成果入选第四届全国教育博览会。招商引资成效显著。持续开展“冬季攻坚行动”，成功签约亿元以上项目168个；“赤子峰会—首届天南地北赤峰人投资创业大会”取得巨大成功，探索实践了招商引资、聚才引智的新模式；先后赴长三角、京津冀、辽沈等地区开展招商洽谈活动，全年协议引进项目投资超千亿元。区域合作不断深化。全面加强与沈阳、朝阳、锦州、承德等地区的战略合作，谋划启动了次临港产业区建设，总投资170亿元的进口资源加工项目签约落地。营商环境持续改善。以打造一流营商环境为目标，出台了深化“放管服”改革的35条具体措施，“八张清单”标准化体系建设全面完成，在全区率先实施了企业全程电子化登记，32项涉企证照事项实现了“多证合一”，行政许可“最多跑一次”事项达到81%；认真落实国家一系列结构性减税降费政策，为企业减免税费36.12亿元。

　　（四）着力改善城乡基础条件。交通项目稳步推进。丹锡高速大板至经棚公路、国道306线林西至大水菠萝一级公路、国道303线凤凰岭至天山一级公路、省道210线新惠至老虎山一级公路等项目建设完成，新投运高速公路113.5公里、一级公路221.5公里。赤峰高铁全线铺轨，玉龙机场改扩建工程完成立项工作。能源水利工程建设加快。朝阳至赤峰天然气长输管道工程获得核准，东台子水库开工建设，琥珀沟水库加快推进，跨区域调水工程前期工作取得重要进展；实施农村牧区饮水安全巩固提升工程425处，受益农牧民10.5万人。新建改造城乡电网1701公里。市政建设成效显著。腾飞大道整体改造工程主线贯通，高铁西站交通枢纽工程全面铺开，环城水系治理工程主体完成，“两山两园”景观提升工程有序推进；新建改造市政管网77.3公里，新增供热面积235万平方米，建设完成公交场站7处、公共厕所88处、垃圾转运站24座，中心城区承载能力和运行保障能力显著提升。乡村振兴战略扎实起步。强力打造7条乡村振兴试验示范带，在24个苏木乡镇实施重点项目132个、完成投资36.5亿元。启动了农村牧区人居环境整治三年行动计划，农村“厕所革命”、垃圾污水无害化处理等试点工作扎实开展，“政府主导、群众主体、社会参与”的农村牧区环境管护长效机制逐步建立。“宽带进乡村”工程完成投资7.5亿元，铺设光缆1.8万公里，惠及农牧民16.4万户。

　　（五）着力补齐民生短板。社保体系兜底能力继续增强。新增城镇就业2.8万人，农牧民转移就业69.3万人，城镇登记失业率始终控制在4%以内，“零就业”家庭保持动态清零；城镇低保标准每人每月提高至635元，农村牧区低保每人每年提高至4455元；全面落实优抚安置政策，接收安置军转干部、退役士兵1767名；完善了脑瘫、自闭症儿童康复补贴政策，养老、医疗、失业、工伤、生育等社保覆盖面不断扩大。教育事业均衡发展迈出新的步伐。中心城区年度投入资金6.7亿元，建成中小学、幼儿园23所，消除“大班额”449个；“全面改薄”工程扎实推进，累计建设校舍54.6万平方米，左旗、翁旗、红山区通过了国家义务教育均衡发展认定；全市高考本科上线率稳居自治区前列。科技服务发展展现新的作为。开发区晋升国家高新区工作已经上报国务院待批，新增自治区级以上创新平台载体14个，争取国家和自治区科技项目123项，申请专利1203件。卫生基础建设取得较大进展。市中心血站迁址新建主体完工，市医院改造提升、传染病医院和蒙医中医医院迁址新建项目加快建设，国家卫生城复审顺利通过。文体惠民工作成效显著。中国契丹辽博物馆、红山文化博物馆、市档案馆完成主体工程，二道井子遗址博物馆开馆运营；全市乌兰牧骑开展惠民演出1418场，市交响乐团正式成立，《乌兰牧骑恋歌》《大漠绿魂》《契丹王朝》等剧作成功首演；举办中国小轮车竞速联赛、全国竞走冠军赛等体育赛事活动500多场。棚户区改造实现重大突破。通过两年努力，基本完成棚户区改造国家计划项目，全市累计实施棚户区改造6.44万套，完成投资564亿元；其中中心城区4.03万套，完成投资329亿元，集中连片的棚户区即将彻底消灭。赤峰监狱搬迁工程具备开工条件。全市民生支出402.5亿元，占一般公共预算支出的78.9%。

　　（六）着力守住公共安全底线。安全生产形势持续好转。针对“责任主体不尽心、安全主体不上心”等主要矛盾，深入开展了“安全生产主体责任落实年”“安全生产警示教育月”活动，“大排查大整治严执法”专项行动取得实效，安全生产事故起数、死亡人数分别下降22.2%和5.4%。食药安全监管全面强化。深入创建国家食品安全示范城市，持续开展食品药品风险隐患排查，切实保障了人民群众饮食用药安全；认真组织开展了疫苗专项排查整治，全市未发现长春长生问题疫苗。社会治理水平不断提升。周密部署扫黑除恶专项斗争，摸排各类涉黑涉恶线索2661条，打掉涉黑组织1个、涉恶团伙89个，破获案件929件，形成了扫黑除恶浓厚氛围。强化网格化管理，社会治理现代化水平进一步提高。高起点、高标准实施了“雪亮工程”，治安防控能力有效提升；智能电视公共法律服务模式荣获“全国智慧司法十大创新案例”，“七五”普法顺利通过自治区中期验收；深入开展信访积案化解集中攻坚活动，一大批“骨头案”“钉子案”得到有效化解，社会大局保持和谐稳定。

　　各位代表，过去的一年里，围绕完成年度目标任务，我们牢固树立“四个意识”，坚持科学依法民主决策，自觉接受人大的法律监督和工作监督，认真执行人大及其常委会的决定，全面接受政协的民主监督和社会各界监督，办理人大代表建议和政协委员提案373件，办结率100%。全面完成第十届嘎查村换届选举工作。严格执行中央八项规定精神和自治区党委、市委配套规定，政府作风进一步好转。民族宗教、国防动员、人民防空、审计统计、粮食安全、气象地震、外事侨务、档案史志等工作成效显著，妇女儿童、慈善、老龄、残疾人等事业取得新的进步。

　　各位代表，看似寻常最奇崛，成如容易却艰辛。在新一届政府的开局之年，我们脚踏实地、砥砺前行，努力兑现“不驰于空想，不鹜于虚声”的承诺，坚持打基础与谋长远有机协同、补短板与稳增长紧密结合、重点工作与中心工作统筹推进，在经济下行压力持续加大的形势下保持经济运行稳中有进，在融资异常困难的条件下有效推进城乡基础建设，在财力十分紧张的情况下实现民生保障较大改善，在矛盾问题复杂交织的背景下促进社会大局和谐稳定，全市经济社会发展朝着全面建成小康社会的目标又迈出了坚实的一步。这些成绩的取得，是习近平新时代中国特色社会主义思想科学指引的结果，是自治区党委、政府关心支持的结果，是市委坚强领导的结果，是市人大、政协监督支持的结果，也是全市上下团结奋斗的结果。在此，我代表市人民政府，向全市各族人民，向给予政府工作大力支持的人大代表、政协委员，向各民主党派、人民团体和各界人士，向各驻赤机构和武警官兵，向所有关心支持赤峰发展的各界朋友，表示崇高的敬意和衷心的感谢!

　　在看到成绩的同时，我们也清醒地认识到，全市发展不平衡不充分的矛盾依然十分突出，制约经济社会发展的一些深层次问题还没得到很好解决。主要表现为：经济总量扩张速度不够快，产业结构调整、转型升级力度不够大；精准脱贫长效机制、生态环保治本机制尚不完善，“三大攻坚战”面临诸多难关；城乡基础设施欠账较多，民生短板仍然突出；新形势下各类矛盾易发多发，公共安全稳定工作面临许多新挑战；部分政府机关工作人员懒政、怠政及腐败现象依然不同程度存在，营商环境亟待进一步优化，等等。对此，我们一定坚持问题导向，采取有力措施切实加以解决。

　　二、2019年工作重点

　　2019年是新中国成立70周年，是决胜全面小康社会、实现第一个百年目标的关键一年，做好今年工作意义重大、影响深远。

　　2019年政府工作总体要求是：以习近平新时代中国特色社会主义思想为指导，按照习近平总书记关于内蒙古工作“四个着力、三个扎实”重要要求，全面贯彻中央经济工作会议和自治区党委十届八次全会暨全区经济工作会议精神，坚持稳中求进总基调，坚持高质量发展要求，坚持以供给侧结构性改革为主线，坚持深化市场化改革、扩大高水平开放，加快建设现代化经济体系，继续打好三大攻坚战，着力激发微观主体活力，统筹推进稳增长、促改革、调结构、惠民生、防风险工作，努力在建设经济大市、人文大市、绿色大市、开放大市、和谐大市上扎实起步，为全面建成小康社会收官打下决定性基础，以优异成绩庆祝中华人民共和国成立70周年。

　　今年全市经济社会发展主要预期目标是：地区生产总值增长6%左右，一般公共预算收入增长6%左右，规模以上固定资产投资增长11%左右，社会消费品零售总额增长7.5%左右，城乡常住居民人均可支配收入分别增长7.5%左右和9%左右，完成国家和自治区下达的其它约束性指标。

　　这些目标的确定，主要是立足赤峰市作为“自治区大市”和“蒙东地区排头兵”的发展定位，既体现了中央经济工作会议坚持高质量发展和稳中求进的要求，又考虑了全市发展实际和实现跨越崛起的战略安排，兼顾客观需要与现实可能，力求达到“跳起来摘桃子”的效果。

　　围绕完成上述目标，今年要重点做好八个方面工作：

　　（一）扎实推进“三大攻坚战”，筑牢全面建成小康社会基础推动防范化解重大风险攻坚战取得阶段性成果。严格落实中央“坚定、可控、有序、适度”的要求，加强政府债务风险评估、预警和监管，规范运作国有投融资平台，按计划进度完成政府债务化解任务，严控政府新增隐性债务。通过市场化手段合理开发利用土地、资产、股权等资源，抓住国家“较大幅度地增加地方政府专项债券规模”的有利时机，加快政府存量债务置换和化解进度，年内化解政府债务110亿元以上，“十个全覆盖”延展工程拖欠工程款全部清零，注意防范“处置风险的风险”。加强地方金融监管能力建设，做好金融重点领域风险防控处置，有效打击违法违规金融活动和恶意逃废债务行为，守住不发生区域性、系统性金融风险的底线。保持房地产市场调控政策的连续性稳定性，合理引导市场预期，持续推动房地产市场健康发展。

　　推动精准脱贫攻坚战取得决定性进展。加大产业项目谋划推进力度，切实破解“有资金、没项目”的难题，实现扶贫产业全覆盖。持续抓好易地扶贫搬迁，强化搬迁人口的产业支撑和就业扶持，确保贫困群众搬得出、稳得住、能致富。认真落实赤峰市健康扶贫22条指导意见，确保完成国家和自治区健康扶贫各项要求。强化扶贫资金管理，创新扶贫项目实施程序，提高资金使用效率。深入开展中央和国家机关定点扶贫工作，加快实施京蒙扶贫协作项目，切实发挥带贫减贫成效。加大对巴林左旗和全市其他深度贫困苏木乡镇的扶持力度，确保这些地区按期实现脱贫退出。建立健全动态监测预警机制，有针对性地对贫困边缘人口进行扶持，及时将新增贫困人口纳入帮扶范围，巩固脱贫攻坚成果。持续加强扶贫领域作风建设，做好中央脱贫攻坚专项巡视反馈问题整改工作。全年投入资金100亿元以上，确保剩余的5.3万贫困人口全部实现稳定脱贫，所有贫困旗县脱贫摘帽。

　　推动污染防治攻坚战取得关键性突破。持续抓好中央自治区环保督察和各类检查反馈问题整改，继续实施大气、水、土壤污染防治12大工程，从源头消灭污染隐患。加快煤烟型大气污染治理、工业废气稳定达标排放、扬尘污染控制等工程，持续改善空气质量，确保中心城区空气质量优良率达到88%以上。深入落实河湖长制，持续推进污水处理、中水回用、水源地保护，全面消除地表水劣Ⅴ类断面，力争国考断面水质优良比例达到66.7%，彻底完成城镇污水处理厂提标改造工作。加大土壤污染治理及潜在风险评估，全面加强有色金属采选企业尾矿库和冶炼企业危险废弃物管理，控制农业面源污染，保持土壤环境质量总体稳定。

　　（二）努力构建现代产业体系，夯实经济高质量发展根基切实提高农牧业现代化水平。继续调整优化种养结构，调减玉米种植30万亩，新建设施农业20万亩，新增优质紫花苜蓿20万亩；突出发展肉牛产业，存栏达到200万头。坚持做大做强龙头企业，提升农牧业组织化水平，市级以上产业化重点龙头企业达到300家，合作组织覆盖率达到57%。推动农牧业与二三产业融合发展，建设一批功能丰富、带动力强的田园综合体。总结推广“敖汉小米”品牌塑造经验，持续打造区域性农畜产品品牌，全市新增“三品一标”认证农畜产品50个。突出抓好重大动物疫病防控，确保畜牧业安全发展。全面启动市农牧业科技园建设，不断提升农牧业科技研发和成果转化能力。

　　切实加快工业经济增量提质步伐。启动“工业经济倍增行动”，集中盘活一批既有工业项目，推动一批重大工业项目建成达产，开工一批新上项目，力争通过3—5年的努力，新增工业产值1000亿元左右，再造一个赤峰工业。加快传统产业转型升级，推动云铜40万吨铜冶炼建成投产、金剑40万吨铜冶炼完成主体工程，力促铜基精深加工、硫酸化工项目实现较大突破；出台推动矿业经济高质量发展的政策，整顿矿业秩序，整合矿产资源，加强矿山绿色化改造，切实推动矿业经济做大做强。力促大唐煤制气工程80万吨甲醇调峰项目、芝瑞抽水蓄能电站项目、安庆工业园区30万吨燃料乙醇项目开工建设，推动能源工业再上台阶。加快培育新兴产业，出台政策措施，依托专业园区、标准化厂房、异地孵化转化等方式，推动生物化工、现代医药、电子信息、新型材料等产业壮大规模、实现集群发展。推动互联网、人工智能与实体经济深度融合，加快传统企业数字化改造。进一步完善工业园区基础设施，提高要素保障能力，放大“飞地经济”政策效应，引导工业项目向高新区和旗县工业园区集中。扎实推进次临港产业区建设，开工建设氧化铝、锂电池正极材料、硅锰合金、钛化工等一批重大项目，加快培育全市工业经济新的增长极。

　　切实推动服务业提档升级。旅游方面，围绕“六张旅游王牌”深度谋划项目、创新招商方式，加快旅游资源整合、并购和重组进程，进一步完善旅游基础设施和配套服务体系，靠项目建设推动旅游资源变成旅游产品，靠配套完善促进旅游产品连成旅游产业；推动玉龙沙湖创建国家5A级景区；借鉴外地经验，举办全市旅游发展大会，以会议促建设，以会议促开发，努力实现全市旅游发展新突破。全年接待游客2100万人次以上、旅游收入350亿元以上。商贸物流方面，抓住我市进入《国家物流枢纽布局和建设规划》的有利契机，加快建设商贸服务型国家物流枢纽承载城市。举办物流产业发展论坛暨招商大会，出台加快现代物流业发展实施意见及优惠政策，健全物流体系，降低物流成本，提高物流效率；持续推动红山物流园区、赤峰商贸物流城建设，整体增强物流业的服务能力和自身竞争力，进一步强化对周边地区的辐射和带动；开工建设赤峰国际物流港，推动赤满欧国际班列常态化运行，尽快实现铁路口岸开放，力争赤峰综合保税区获得国家批准；推动维多利摩尔城、桥北新众联等重点商业项目开业运营，完成大福市场、千禧市场退城入园，加快赤峰高铁站周边精品商务区、松北新城商业综合体建设，做大做强蒙东医药物流园；大力实施基层供销合作社改造提升工程，推动传统商贸物流业线上线下融合发展，启动国家级跨境电子商务综合试验区创建工作。全面落实粮食安全行政首长负责制。金融方面，进一步优化金融环境、丰富金融业态、创新服务模式，切实提高服务实体经济能力；启动塞北金融小镇二期工程建设，吸引更多金融、类金融机构入驻，种好这块地方金融发展的“试验田”；继续推进“企业挂牌上市四年行动计划”，重点实施民营企业股份制改造集中行动。积极培育教育、育幼、养老、医疗、文化等新型服务业，开拓更多消费渠道，在提升群众生活质量的基础上拉动经济增长。大力发展信息科技产业，启动松山信息科技产业园区建设（三）深入实施乡村振兴战略，开创“三农三牧”工作新境界坚持规划引领，明确努力方向。以《赤峰市关于实施乡村振兴战略的意见》和《全市乡村振兴战略规划（2018-2022）》为引领，紧扣“产业兴旺、生态宜居、乡风文明、治理有效、生活富裕”20字方针，指导各地区因地制宜地编制好具体规划。分地区、分部门细化实化工作重点，分领域制定专项实施方案，明晰年度重大计划、重大工程、重点项目的时间表、路线图，确保各项工作有的放矢、有序推进。

　　突出工作重点，解决关键问题。注重特色、集中布局，培育发展富民产业，夯实乡村振兴基础。积极推进“四好农村路”建设，全面完成“宽带进乡村”工程，深入推进电信普遍服务4G基站建设试点，持续改善农村牧区基础条件。重点推进村容村貌提升、垃圾污水治理、“厕所革命”等工程，深入实施农村牧区人居环境整治三年行动计划。注重发挥乡贤作用，推进农村牧区社会治安综合治理，进一步健全法治、德治、自治相结合的治理体系，推广“一村一警务助理”模式，加强乡村治理体系创新。

　　抓好示范带动，加强探索创新。继续整合项目资金集中投入，支持7个旗县区建设试验示范带，支持其他旗县建设试验示范点，逐步扩点带面，提高乡村振兴的成效。年内计划整合投入资金78.3亿元，实施重点项目194个，探索积累不同地区、不同领域乡村振兴的模式路径。

　　（四）大力加强市政建设和管理，提高城镇宜居水平大力提升中心城区承载能力。加大基础设施补短板力度，加快中环路快速化改造，完成腾飞大道整体改造及大板路南通工程，打通宁澜路、松州路等干线通道，从根本上改善市区交通条件。着力提升水资源保障能力，完成环城水系治理、三座店水库向中心城区引供水工程，开工建设阴河新水源地工程，新建再生水管网96公里。加强城区绿化美化，完成“两山两园”景观绿化提升工程，谋划西山生态公园开发建设，进一步提升城市品质。启动中心城区新增热源论证工作。新增标准化公厕90座。有计划、有步骤推进老城区改造，围绕管网更新、交通疏堵、空间增绿、风貌治乱、业态提升等重点领域，继续实施一批改造工程。加大老旧小区改造力度，上半年完成棚户区改造全部尾留任务，显著提升城市宜居水平。在深入挖掘提炼的基础上，精心策划论证城市景观嵌入工程，适度有序植入特色文化元素，彰显城市风格，增强城市辨识度。

　　大力提升小城镇建设水平。坚持量力而行、尽力而为，高标准建设改造城关镇，“面子”“里子”一起抓，加快完善基础设施，提升服务功能，促进产城融合，提高城关镇的辐射带动力。其他重点小城镇要突出“雪中送炭”，以供水、排水、供热、垃圾和污水处理为重点，加快补齐市政基础的突出短板，有效提升承载能力和集聚功能。

　　大力提升城市精细化管理水平。深入推进城市管理执法体制改革，建立完善城市综合管理体系，推动城市管理服务标准化。巩固国家卫生城成果，深入开展新时期爱国卫生运动，推动市容市貌环境持续好转。加快推进城市信息化管理步伐，提高智慧化、精细化管理水平。通过市场化方式推进环卫一体化、市政设施维护和园林绿化养护，年内机械化清扫率达到70%。以创建国家卫生县城、国家园林县城为目标，提高城关镇综合管理水平。

　　（五）持续改善基础条件，增强经济社会发展后劲加快完善立体化交通网络。推进铁路重点项目建设，力促赤峰高铁进入联调联试阶段，争取集通铁路、叶赤铁路扩能改造和京通铁路电气化改造工程建成完工，推动集通铁路电气化改造工程开工建设，加快推进大板铁路枢纽前期工作。抓好公路重点项目建设，推动国道306线十家满族乡至牛家营子一级公路、国道305线玛尼罕至玉田皋二级公路、省道210线高日罕至羊场二级公路建成通车，加快推进丹锡高速经棚至乌兰布统公路建设，力促国道508线小城子至平泉一级公路、国道305线林东至下伙房一级公路、省道210线巴拉奇如德至高日罕二级公路开工建设，争取乌丹至天山高等级公路具备开工条件。积极构建航空网络体系，开工建设玉龙机场改扩建工程，争取林西支线机场获得立项批复，推动克旗乌兰布统通用机场开工建设、阿旗通用机场正式通航，做好松山区航空小镇项目前期工作，加快形成通用航空网络。

　　加强水利能源等基础设施建设。加快推进东台子水库、琥珀沟水库建设，力促跨区域调水工程获批并具备开工条件。开工建设并完成朝阳至赤峰燃气管道工程。积极寻求赤峰清洁能源输出基地前期工作取得突破。继续改造提升城乡电网，开工66千伏及以上线路473公里，完成17项重点输变电工程。加快推动信息通信基础设施建设，全面完成城乡光纤改造，实现4G移动网络城市地区深度覆盖、农村牧区逐步覆盖，移动宽带用户普及率达到80%。

　　加大生态保护修复力度。强化国土纵深自然修复，实行最严格的生态环境管理制度，认真执行《赤峰市禁牧休牧和草畜平衡条例》，全面落实草原生态奖补政策，加快恢复森林草原湿地生态系统。搞好重点区域工程治理，持续实施京津风沙源治理、天然林保护、退耕还林还草、已垦草原治理试点等重点生态工程，全年完成林业生态建设60万亩、水土保持治理138万亩；实施通道绿化提质工程，用两年时间集中提升990.8公里重要通道绿化品质，今年完成816.8公里；开工建设克旗规模化林场。加强水资源保护，深入落实《赤峰市地下水保护条例》，全部关停中心城区自备水源井，通过法规约束、市场化调节等方式，不断提升城乡居民节水意识。按规划实施老哈河上游生态保护与环境整治“三年行动计划”，年内完成投资36亿元以上。全面推进达里诺尔流域综合整治工程，年内完成投资1.1亿元。持续推进绿色矿山建设，创建绿色矿山27家。

　　（六）全面深化改革扩大开放，激发创新发展活力和动力积极推动各领域改革。结合新一轮政府机构改革，进一步优化职能配置、理顺职责关系，加快构建系统完备、科学规范、运行高效的机构职能体系，配套建立生态环保、文化市场、交通运输、农牧业、城市管理等综合执法体系。深入推动农村牧区综合改革，全面完成农村牧区承包土地确权登记颁证工作，持续推进土地草牧场“三权分置”改革和农村牧区集体产权制度改革。加快职业教育综合改革试验区建设，统筹推进投融资体制、财税管理体制、资源市场化配置等重点领域改革。

　　强力推进招商引资。扎实开展“冬季攻坚行动”，认真落实各项奖励补助政策和考核问效要求，确保上半年引进亿元以上项目100个以上，实现实质性落地开工。创新招商引资模式，加快推广标准化厂房招商，确保年内建成标准化厂房50万平方米左右。积极构建全社会招商格局，通过政府授权和购买服务等方式，委托地区商会、行业协会和社会组织招商，对贡献突出的企业、专业机构和社会人士给予重奖，充分调动各方面参与招商的积极性。深度策划办好第二届“赤子峰会”，建立全天候、常态化联络机制，举办系列论坛活动，吸引天南地北赤峰人回乡投资兴业。高水平开展好新一轮京津冀、长三角、珠三角地区招商推介活动，力争达成产业链、园区层面的合作，引进一批整体转移项目，加快创建国家级承接产业转移合作示范区。

　　深入开展区域经济合作。切实做好“依托京津”的文章，持续加强与北京市全方位合作，主动承接非首都功能产业转移，深化与承德市战略合作，加强公路互通和资源共享，推动两地旅游合作开发整体升级。实质突破“借力辽沈”的课题，密切与沈阳、锦州、朝阳等地合作，加强公路、铁路等基础设施互联互通，深入开展装备制造、能源化工、港口贸易、文化旅游等领域产业协作，厚植参与东北经济区域合作的优势。抢抓国家“一带一路”建设的机遇，协同推进中蒙俄国际铁路新通道建设，加快建设进出口贸易加工区和国际物流枢纽城市，通过借船出海增添地区发展活力。

　　背水一战改善营商环境。切实增强优化营商环境的紧迫感和危机感，在“放管服”改革中采取更多“革命性”措施，推动营商环境彻底改善。深化“马上办、网上办、就近办”改革，实行一门受理、只跑一次和部门代办制度，推行联合审批、多图联审、区域审批，加快实现政务服务一网通办。全面推行公共资源交易电子化，坚决破除地方政策壁垒。稳步推开“证照分离”改革，有序推进“多证合一”改革，积极开展“政银合作”，实现企业登记与银行开户网上联办，进一步压缩企业开办时间，努力营造更加宽松便捷的市场准入环境。

　　综合施策激发民营经济活力。全面落实自治区促进民营经济高质量发展的26条措施，推动民营经济繁荣振兴。充分利用各级民营经济及中小企业发展纾困基金，积极引导企业实施债转股，鼓励企业多渠道直接融资。严格落实国家减税降费政策，积极争取要素资源优惠政策，努力降低民营企业运营成本。认真执行公平竞争审查制度，全面兑现招商引资优惠政策，限时清偿政府和国有企业拖欠民营企业账款，以政府诚信提振民间投资和民营企业发展信心。推进“个转企、小升规、规改股、股上市”，在培育铺天盖地的中小微企业的同时，努力培育更多顶天立地的大型企业和开天辟地的创新型企业。扎实开展第四次全国经济普查工作。

　　（七）切实加大民生社会事业补短板力度，不断增进民生福祉促进社会就业更加充分。突出做好高校毕业生、农村牧区转移劳动力、退役军人等重点群体就业工作，做好城镇就业困难人员和登记失业人员等帮扶工作，加大农民工工资清欠力度，确保完成军转干部、退役士兵安置任务，城镇新增就业2.3万人，农牧民转移就业68.5万人，登记失业率控制在4%以内。

　　促进教育发展更加均衡。深入落实《关于学前教育深化改革规范发展的若干意见》，大力实施第三期学前行动计划，年内新建幼儿园34所，普惠性学前教育资源覆盖率达到70%。加快义务教育均衡发展，年内新建、改扩建义务教育学校13所，消除“大班额”286个，确保敖汉旗通过国家义务教育均衡发展认定。实施高中攻坚计划，推进普通高中优质特色多样化发展。实施民族特色学校建设工程，确保新城蒙小年内交付使用。推动职业教育差异化发展，注重打造职业教育特色品牌，加快推进产教融合、校企合作。积极筹建赤峰应用职业技术学院，大力支持赤峰学院改善办学条件。

　　促进医疗服务更加便民。深化医药卫生体制改革，进一步完善分级诊疗制度，做实做细家庭医生签约服务，制定基本医疗个性化签约服务包。积极推进医疗卫生信息化，建设全民健康信息“三库一卡”。加强基本公共卫生服务和预防保健工作，有效防控重大疾病发生。完善落实全面两孩政策体系，促进人口均衡发展。强力推进医疗服务能力提升工程，完成市医院改造提升、传染病医院和蒙医中医医院迁址新建主体工程，建成投运市中心血站。

　　促进社会保障更加健全。深入实施全民参保计划，持续扩大社会保险覆盖面，完善社会保障制度，加强社保基金管理，按要求推动养老保险基金、工伤保险基金实现自治区级统筹。提高城乡低保、特困供养标准，健全社会救助与物价上涨联动机制。开展养老院服务质量建设专项行动，加大苏木乡镇敬老院整合力度，鼓励社会力量参与养老服务，推进医养结合和老龄健康工作。加强残疾人康复服务，维护妇女儿童合法权益，大力发展人道事业和公益性慈善事业。

　　促进社会各项事业更加繁荣。加快以企业为主体的科技创新体系建设，建设自治区级以上创新平台载体15家，办好2019赤峰·中国北方农业科技成果博览会。加快引进高层次急需人才，为推动经济高质量发展提供智力保障。大力实施文化惠民工程，持续推进乌兰牧骑精品文艺创作，确保完成公益性演出1000场以上。做好全国第十四届冬运会高山滑雪项目承办准备工作，积极办好特色体育赛事和文化节庆活动，推进市档案馆建成投入使用。以史前文化和契丹辽文化为重点，系统启动特色文化品牌塑造工程，完成内蒙古史前文化博物馆建设，加快推进中国契丹辽博物馆、红山文化博物馆建设，加快兴隆洼国家考古遗址公园、辽上京国家考古遗址公园建设，持续推进红山文化、辽上京和祖陵遗址申遗工作。全面开工赤峰监狱搬迁工程。

　　（八）全力抓好公共安全工作，维护和谐稳定社会大局坚决做好安全生产工作。全面落实安全生产责任制，切实压实属地责任、行业部门监管责任和企业主体责任。实施“安全生产改革年”行动，分行业制定企业安全风险防范模块，聘请行业专家和第三方机构定期分析研判重点行业领域安全生产状况，全面提高政府监管效能，从根本上解决主体责任不落实问题，坚决防范遏制重特大事故发生。深入开展重点行业领域安全生产专项整治工作，从严打击违法违规生产经营企业。加强安全生产宣传培训，全面开展警示教育，深入开展安全生产“七进”活动，加强逃生避险、自救互救技能培训，不断提高全社会安全意识和安全素质。

　　持续加强市场监督管理。深入推进市场监管综合行政执法改革，进一步整合市场监管职能，有效提高监管执法效能。全面推行“双随机、一公开”监管，强化反垄断和反不正当竞争执法，积极营造公平竞争的市场环境。开展“诚信赤峰”行动，搭建全市统一征信平台，全面加强信用信息的采集、共享、使用，实现守信联合激励、失信联合惩戒，加快改善社会信用环境。全面实施食品药品安全战略，持续推进国家食品安全示范城市创建工作，完成食品小作坊加工园区、标准化农贸市场建设任务，严肃查处食品药品领域违法行为，确保不发生区域性、系统性食品药品安全事件。

　　加快构建共建共治共享社会治理格局。深入开展扫黑除恶专项斗争，针对重点案件、重点问题、重点地区集中攻坚，深入实施打伞破网行动，彻底铲除黑恶势力滋生土壤。继续加强平安赤峰建设，系统强化治安隐患清理，全面完成“雪亮工程”，努力提高公安工作智能化、法治化、规范化、专业化水平。加大信访法治化建设力度，认真学习推广“枫桥经验”，及时回应群众合理诉求，严控信访增量，减少信访存量。加强互联网安全管理，打造清朗网络空间。扎实推进民族团结和边疆稳固，深入开展民族团结进步创建工作，推动民族团结进步事业走在全区前列。扎实开展国防动员准备，严格执行人防工程建设标准，完成国家重要经济目标防护体系建设试点任务。持续做好应急救灾避险演练，综合提高全社会防灾减灾能力。加快构建多元化基层社会治理模式，支持工会、共青团、妇联、红十字会等人民团体广泛参与社会治理，充分发挥社会组织在社会管理和公共服务中的作用。

　　各位代表！我们深知，建设“五个大市”，任务艰巨，使命光荣，既考验着政府的攻坚能力，也对政府自身建设提出了更高要求。在新的一年里，我们将更加注重忠诚与担当，持续推动政府政治建设。牢固树立“四个意识”，坚定“四个自信”，坚决做到“两个维护”，始终在思想上行动上同以习近平同志为核心的党中央保持高度一致，用贯彻落实党中央各项决策部署的具体实践来诠释绝对忠诚。以赤峰发展为己任，不回避矛盾，不贪图虚名，顶着压力干，迎着困难上，切实担负起推动高质量发展的责任与担当。我们将更加注重民主与法治，持续促进政府科学决策。坚决落实市人大及其常委会各项决议决定，自觉接受人大法律监督、工作监督；认真听取各民主党派、工商联、无党派人士和人民团体的意见建议，主动接受政协民主监督；及时向社会各界公开重大决策事项及其实施情况，自觉接受各方面的批评。大力推进依法行政，把法治精神、法治思维、法治方式融入政府工作全过程。我们将更加注重实干与效率，持续提高政府执行能力。坚持以目标倒逼过程，实行政府重点工作上台账、列清单、定时限，努力提高工作实效。坚持干字当头、实字为先，盯大事、盯发展、盯项目，以实实在在的工作成效树立形象、赢得信任。我们将更加注重清廉与公正，持续推进政府作风建设。认真落实全面从严治党要求，严格执行改进作风的各项规定，下力气从制度和机制上寻找和堵塞腐败漏洞，通过专项行动深入整治“懒官、庸官、乖官、巧官”问题，努力实现干部清正、政府清廉、政治清明。

　　各位代表！站在建设“五个大市”的新方位，赤峰发展的基础日益雄厚，跨越崛起的势头愈发强劲。让我们更加紧密地团结在以习近平同志为核心的党中央周围，在自治区党委政府和市委的坚强领导下，以时不我待、只争朝夕的精神状态，抢抓机遇，坚定信心，锐意进取，攻坚克难，奋力谱写赤峰跨越崛起的新篇章，以优异成绩庆祝中华人民共和国成立70周年!
